# Audit & Reaudit of Assessments Regarding Substance Misuse in Patients Referred to Liaison Psychiatry Service

**DOI:** 10.1192/bjo.2022.488

**Published:** 2022-06-20

**Authors:** Oriyomi Shittu, Samlan Mushtaq, Dervis Maria

**Affiliations:** 1 Livewell Southwest, Plymouth, United Kingdom; 2 Derriford Hospital, Plymouth, United Kingdom

## Abstract

**Aims:**

1- What percentages of people presenting to general hospital that are referred to Liaison Psychiatry service have Substance misuse problems? 2- Are the assessments by Liaison Psychiatry services identifying substance misuse problems? 3- If substance misuse problem is identified then are we offering any advice/intervention/referral

**Methods:**

To look at 100 consecutive assessments by using an audit proforma to capture information required to answer above questions.

**Results:**

In 78% of cases there was evidence documented that the patient was asked about alcohol use. In 22% - no evidence patient was asked about alcohol use.

- 77% documentation about drug use. 23% no evidence documented that the patient was asked.
- Of those asked about their alcohol use (n = 62), a misuse problem was identified by clinicians making the assessment in 6 cases (10% of those asked).
- Of those asked about their drug use (n = 61), a misuse problem was identified by clinicians making the assessment in 8 of cases (13% of those asked).

Of those with a substance misuse problem identified (n = 15), 20% identified misuse of both alcohol and drugs, 40% identified misuse of alcohol only, and 40% identified misuse of drugs only.

- Of those with a substance misuse problem identified (n = 15), 73% were offered advice or an intervention, and 27% had no intervention documented.

**Conclusion:**

Just over a fifth of patients assessed were not asked about alcohol or drug use. This has improved since August 2020 when nearly half of the patients assessed were not asked about alcohol or drug use.

- Since audit in August 2020, there has been a 21% increase in documentation of advice or intervention being offered to patients identified to have a substance misuse problem.

